# Tracking the stochastic fate of cells of the renin lineage after podocyte depletion using multicolor reporters and intravital imaging

**DOI:** 10.1371/journal.pone.0173891

**Published:** 2017-03-22

**Authors:** Natalya V. Kaverina, Hiroyuki Kadoya, Diana G. Eng, Michael E. Rusiniak, Maria Luisa S. Sequeira-Lopez, R. Ariel Gomez, Jeffrey W. Pippin, Kenneth W. Gross, Janos Peti-Peterdi, Stuart J. Shankland

**Affiliations:** 1 Department of Medicine, Division of Nephrology, University of Washington, Seattle, WA, United States of America; 2 Department of Physiology & Biophysics, Zilkha Neurogenetic Institute, Keck School of Medicine, University of Southern California, Los Angeles, CA, United States of America; 3 Department of Nephrology and Hypertension, Kawasaki Medical School, Kurashiki, Japan; 4 Department of Molecular and Cellular Biology, Roswell Park Cancer Institute, Buffalo, NY, United States of America; 5 Department of Pediatrics, University of Virginia School of Medicine, Charlottesville, Virginia, United States of America; University of Houston, UNITED STATES

## Abstract

Podocyte depletion plays a major role in focal segmental glomerular sclerosis (FSGS). Because cells of the renin lineage (CoRL) serve as adult podocyte and parietal epithelial cell (PEC) progenitor candidates, we generated *Ren1cCre/R26R-ConfettiTG/WT* and *Ren1dCre/R26R-ConfettiTG/WT* mice to determine CoRL clonality during podocyte replacement. Four CoRL reporters (GFP, YFP, RFP, CFP) were restricted to cells in the juxtaglomerular compartment (JGC) at baseline. Following abrupt podocyte depletion in experimental FSGS, all four CoRL reporters were detected in a subset of glomeruli at day 28, where they co-expressed de novo four podocyte proteins (podocin, nephrin, WT-1 and p57) and two glomerular parietal epithelial cell (PEC) proteins (claudin-1, PAX8). To monitor the precise migration of a subset of CoRL over a 2w period following podocyte depletion, intravital multiphoton microscopy was used. Our findings demonstrate direct visual support for the migration of single CoRL from the JGC to the parietal Bowman’s capsule, early proximal tubule, mesangium and glomerular tuft. In summary, these results suggest that following podocyte depletion, multi-clonal CoRL migrate to the glomerulus and replace podocyte and PECs in experimental FSGS.

## Introduction

Adult podocytes are terminally differentiated glomerular epithelial cells that form the outer layer of the glomerular filtration barrier and are unable to self-replicate [1]. As a result, a major limitation in their recovery and repair process in many disease states is their inability to restore their numbers following depletion [2, 3]. Once total podocyte number decreases below a certain threshold in glomerular disease, glomerular scarring ensues [4–6]. For these reasons, recent studies have been devoted to trying to discover how adult podocytes can be replaced from other sources.

Two adult podocyte progenitor candidates residing in the kidney have been identified, namely glomerular parietal epithelial cells (PECs) [7–11] and cells of renin lineage (CoRL) [7, 12, 13]. We and others have shown that CoRL have marked cell plasticity properties [14] in that they can during development and under different conditions, lose their endocrine and/or contractile functions and de-differentiate into a variety of different adult cell types [15]. These include mesangial cells [10, 16–18], pericytes [10, 13, 19, 20], vascular smooth muscle cells [10, 13], EPO-producing cells [21], hematopoietic-immune-like cells [14], glomerular parietal epithelial cells [8, 10, 12], and podocytes [7, 8]. However, in all circumstances, the clonal properties of CoRL progenitors has not been reported. Although we have employed state of the art fate mapping techniques that temporally and permanently label specific cohorts of cells, additional proof of cell migration from the juxta- to the intra-glomerular compartment was needed.

The purposes of the current studies was twofold: first, RenCre confetti reporter mice were used to determine the clonality of CoRLs that begin to express podocyte and PEC markers in the setting of abrupt podocyte depletion. Second, live imaging of the same glomeruli in the same intact kidney over several days was used to track the migration of labeled CoRL from the juxta-glomerular compartment to the intra-glomerular compartment.

## Methods

### Cells of renin lineage confetti reporter mice

#### Ren1cCre/R26R-ConfettiTG/WT

In order to study the potential clonality of cells of renin lineage (CoRL), *Ren1cCre* mice described previously [22] were crossed with commercially available *Confetti (Gt(ROSA)26Sortm1(CAG-Brainbow2*.*1)Cle/J)* mice from The Jackson Laboratory (Bar Harbor, ME). One of the four fluorescent reporters (CFP, RFP, YFP, GFP) is stochastically and constitutively expressed per allele following transient Cre-mediated recombination. PCR was performed on tail biopsies to confirm *Ren1cCre-Confetti* genotypes [22, 23]. Twelve 8–10 weeks old double transgenic mice (heterozygous for Cre and Confetti) were used to permit cell specific assessments of the mobilization of CoRL in an inducible model of FSGS (see below). Mice were housed in the animal care facility of the University of Washington under specific pathogen-free conditions with ad libitum food and water. Animal protocols were approved by the University of Washington Institutional Animal Care and Use Committee (2968–04).

#### Ren1dCre /R26R-ConfettiTG/WT

In order to perform intravital serial multiphoton microscopy, female *Ren1d-Confetti* mice 4–6 weeks of age were generated by intercrossing mice expressing the floxed *R26R-Confetti* construct [24] (purchased form The Jackson Laboratory, Bar Harbor, ME, USA) and *Ren1d-Cre* mice [11]. These mice feature the expression of membrane-targeted CFP, nuclear GFP, cytosolic YFP or cytosolic RFP in cells of the renin lineage in the same fashion and distribution as the *Ren1cCre/R26R-ConfettiTG/WT* mice above. All animal protocols were approved by the Institutional Animal Care and Use Committee at the University of Southern California.

### Abdominal imaging window

Surgical implantation of a dorsal abdominal imaging window (AIW) above the left kidney was performed on *Ren1d-Confetti* mice using aseptic surgical procedures as described recently [25]. This approach represents a refinement of our recently developed technique [26], and allows long-term, non-invasive imaging of the kidney. Briefly, the animals were anesthetized with a combination of ketamine (100 mg per kg body weight) and xylazine (10 mg per kg body weight). The AIW consists of a re-usable titanium ring (kind gift from Ina Schiessl, University of Regensburg, Germany) with a 1-mm groove on the side and a 170 μm thick coverslip fixed on the top. The glass coverslip was secured using tissue glue. After preparation of the window, the AIW was surgically implanted into the flank incision on the dorsal skin and abdominal wall right above the left kidney, and held in position by a purse-string suture, which was concealed within the groove of the AIW ring to prevent mice from biting or pulling the sutures. To further reduce the risk of dislodgement of the AIW, a nonwoven non-resorbable suture (Prolene, Somerville, NJ, USA) was used, which can be tightened once the window is inserted and which can keep the window secured for >5 weeks. Postoperative care included animal monitoring to ensure that vital signs and body weight returned to preoperative status.

### Inducing experimental FSGS typified by abrupt podocyte depletion

Experimental focal segmental glomerulosclerosis (FSGS) was induced in *Ren1cCre /R26R-ConfettiTG/WT* and *Ren1dCre/R26R-ConfettiTG/WT* mice strains with a cytotoxic sheep anti-podocyte antibody, as previously described [22, 27, 28]. This cytotoxic antibody induces abrupt podocyte depletion, accompanied by glomerulosclerosis. Adult mice were given 2 doses of sheep anti-glomerular antibody at 12mg/20g body weight via IP injection, 24 hours apart. Thereafter, the experimental protocols differed by strain to address different questions as follows:

*Ren1cCre/R26R-ConfettiTG/WT* mice were sacrificed randomly on day 14 (n = 6) and on day 28 (n = 7) of disease. Uninjected mice (n = 4) served as baselines. At sacrifice, mice were perfused with 10ml of ice cold PBS to remove excess red blood cells. Kidneys were split in half and one half was fixed overnight at 4°C in 10% neutral buffered formalin (Globe Scientific, Paramus, NJ, USA), rinsed in 70% Ethanol, processed and embedded in paraffin. The other half was fixed for 45 minutes in 4% Paraformaldehyde Solution (PFA) in PBS (Affymetrix, Santa Clara, California, USA), washed in 30% sucrose overnight at 4°C, patted dry, rinsed briefly in OCT, embedded in OCT, and frozen in a dry ice 100% ethanol bath. Sections were then cut from the paraffin or OCT blocks at 4μm thickness.

*Ren1dCre/R26R-ConfettiTG/WT* mice were used to assess the migration of CoRL from the juxta-glomerular compartment to the intra-glomerular compartment by intravital serial multiphoton microscopy (see below for details).

### Intravital serial multiphoton microscopy (MPM)

Under continuous anesthesia (Isoflurane 1–4% inhalant via nose-cone), *Ren1dCre /R26R-ConfettiTG/WT* mice with the AIW above were placed on the stage of the inverted microscope as described previously [29]. Body temperature was maintained with a homeothermic blanket system (Harvard Apparatus, Holliston, MA, USA). Alexa 594 bovine serum albumin was injected iv to label the vasculature. The images were acquired using a Leica TCS SP5 multiphoton confocal fluorescence imaging system with a 63× Leica glycerol-immersion objective (numerical aperture (NA) 1.3) powered by a Chameleon Ultra-II MP laser at 860 nm (Coherent) and a DMI 6000 inverted microscope’s external nondescanned HyD detectors (Leica Microsystems, Heidelberg, Germany). Short-pass filters (680 nm for blue and red and 700 nm for green and yellow), dichroic mirrors (cut off at 515 nm for green and yellow and at 560 nm for blue and red) and bandpass filters were specific for detecting CFP, GFP, YFP and RFP emission (473, 514, 545 and 585 nm, respectively) (Chroma, Bellows Falls, VT, USA). The potential toxicity of laser excitation and fluorescence to the cells was minimized by using a low laser power and high scan speeds to keep total laser exposure as minimal as possible. The usual image acquisition consisted of only one z stack per glomerulus (<3 min), which resulted in no apparent cell injury. Serial imaging of the same glomerulus in the same animal/kidney was performed once every 3 days for up 12 days after the first imaging session.

### BrdU/FDU labeling of mice to assess proliferation

Amersham Cell Proliferation Labeling Reagent (GE Healthcare Life Sciences, Little Chalfont, UK) was administered to quantitate cell proliferation. Mice were given 3 consecutive IP injections of 10ul per gram body weight of 5-bromo-2-deoxyuridine and 5-fluoro-2’-deoxyuridine as recommended by the manufacturer, with the first dose given the day after the last dose of antibody. BrdU immunostaining was performed on paraffin embedded tissue that was processed as described above and incubated at 4°C overnight with a primary anti-BrdU antibody (1:200, GE Healthcare Life Sciences, Little Chalfont, UK), and then with biotinylated anti-mouse IgG secondary (1:500, Vector Laboratories, Burlingame, CA, USA) followed by streptavidin-conjugated Alexa Fluor 594 (1:100, Molecular Probes, Eugene, OR, USA). FITC 488 conjugated anti-GFP antibody, which binds to all four of the confetti variants (Rockland Immunochemicals for Research, Gilbertsville, PA, USA) was used to detect all four confetti reporters at the 488nm wavelength channel (GFP).

### Visualization of confetti reporter in *Ren1cCre /R26R-ConfettiTG/WT* mice

Immunostaining was examined on a Leica TCS SPE II laser scanning confocal microscope (Solms, Germany) with X40 (1.3 NA) or X60 (1.45 NA) oil objectives. In order to visualize the multicolor confetti reporter, four-μm cryo sections were rinsed in PBS (pH 7.4) to remove OCT compound (VWR, Radnor, PA, USA) and mounted with vectashield (Vector Labs, Burlingame, CA, USA). Confocal images were acquired in 1,024 X 1,024 pixel format with 8 bit intensity resolution. The acquisition was set in the green, red, yellow and cyan wavelengths as follows; CFP 458 nm excitation, 464–495 nm emission, GFP 488 nm excitation, 497–510 nm emission, YFP 514 nm excitation, 517–540 nm emission, and RFP 561 nm excitation, 575–654 nm emission.

### Assessment of glomerular injury and podocyte density

In both reporter mouse strains, immunostaining was performed for p57 with Periodic Acid Schiff (PAS) counterstaining to measure podocyte number and assess glomerulosclerosis. In brief, paraffin sections were processed as described above, with antigen retrieval in 1 mM EDTA, pH 8.0. Endogenous peroxidase activity was quenched with 3% hydrogen peroxide and sections were incubated overnight at 4°C with a primary rabbit anti-p57 (1:800, Santa Cruz Biotechnology, Santa Cruz, CA,USA) followed by rabbit on rodent HRP-polymer (Biocare Medical, Concord, CA, USA). Visualization of immunostaining was by precipitation of diaminobenzidine (DAB; Sigma-Aldrich, St. Louis, MO, USA). Counterstaining was performed with Periodic Acid Schiff (PAS) by washing slides in fresh 0.5% periodic acid (Sigma-Aldrich, St Louis, MO, USA) for 8 minutes, washed for 5 minutes in ddH2O, sections were incubated for 10 minutes at room temperature with Schiff’s Reagent (Sigma-Aldrich, St Louis, MO, USA), washed 2x for 5 minutes in fresh 0.5% sodium metabisulfate (Sigma-Aldrich, St Louis, MO, USA), and washed for 5–10 minutes under running tap water. Slides were dehydrated in ethanol and mounted with Histomount. To measure podocyte density we used the correction factor (CF) method previously reported by Venkatareddy *et al* [30].

### Identifying podocytes in *Ren1cCre /R26R-ConfettiTG/WT* mice reporter mice

Indirect immunofluorescence staining was performed on 4μm tissue sections from mouse renal biopsies fixed in 4%PFA as described above. Frozen sections were thawed from -80^o^ C storage and allowed to air-dry. All sections were equilibrated in PBS-buffered saline (pH7.4) then blocked with Background Buster (Accurate Chemical & Scientific Corporation, Westbury, NY, USA) for 30 minutes to minimize nonspecific protein interactions. Endogenous biotin activity was quenched with the Avidin/biotin blocking kit (Vector Laboratories, Burlingame, CA, USA). After blocking, tissue sections were incubated overnight at 4°C with the appropriate primary antibodies: guinea pig antibody to nephrin (Fitzgerald Industries International. Inc., Concord, MA, USA), mouse antibody to synaptopodin (Fitzgerald Industries International. Inc., Concord, MA, USA), rabbit antibodies to podocin (Abcam, Cambridge, MA, USA) and rabbit antibody to p57 (1:800, Santa Cruz Biotechnology, Santa Cruz, CA, USA). The appropriate biotinylated secondary antibody (Vector Laboratories) was applied followed by Streptavidin, AlexaFluor 647 conjugate (Life Technologies—Molecular Probes, Grand Island, NY, USA). All immunofluorescence samples were mounted using Vectashield mounting medium with DAPI (Vector Labs, Burlingame, CA, USA). A fluorescein isothiocyanate (FITC) conjugated anti-GFP antibody, which binds all four of the confetti variants (1:100, Rockland Immunochemicals for Research, Gilbertsville, PA, USA) was used to detect all four confetti reporters at the 488nm wavelength channel (FITC). As a negative control, all staining was performed without primary antibodies.

### Statistical analysis

The results are expressed as mean ± SEM. Groups were compared using a one-way or two-way ANOVA for multiple comparisons with Bonferroni post hoc analysis with significant set at p<0.05.

## Results

### Abrupt podocyte depletion in *Ren1cCre/R26R-ConfettiTG/WT* mice is followed by partial replacement by another cell type

**Fig 1:**
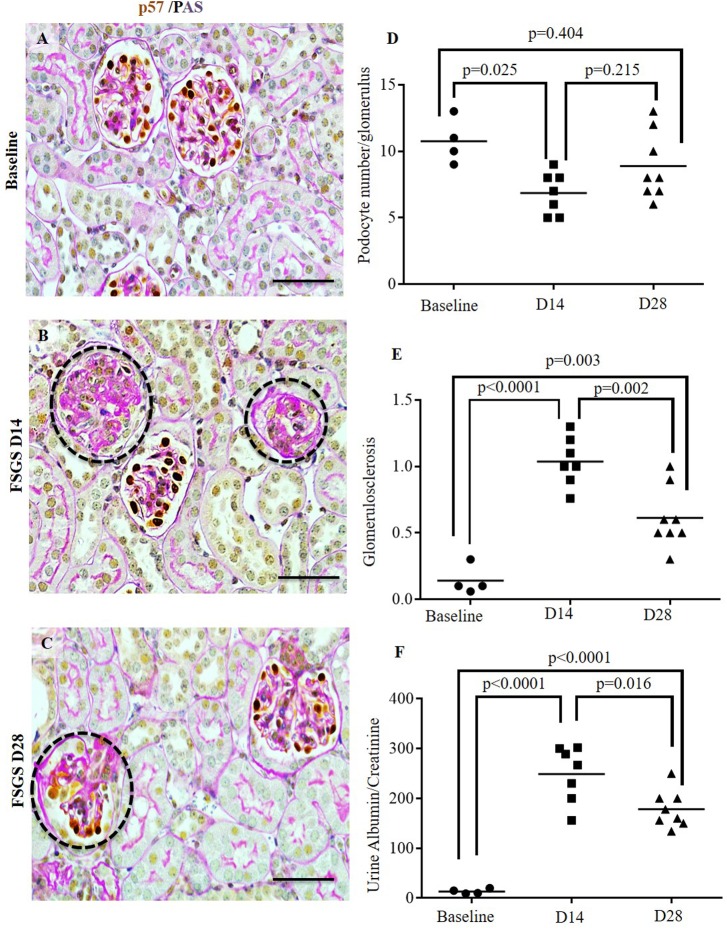
Partial podocyte replacement in *Ren1cCre /R26R-ConfettiTG/WT* mice with experimental FSGS. (A-C) Double staining was performed for the podocyte marker p57 (brown, nuclear) and counterstain with Periodic acid Schiff's (pink stains matrix, blue stains nuclei) in reporter mice at baseline (A), day 14 (D14) FSGS (B) and D28 FSGS (C). (D) Podocyte number was lower at D14 compared to baseline, and partial recovered by D28. (E) Glomerulosclerosis was highest at D14 FSGS, with a significant reduction by D28. (F) The urinary albumin to creatinine ratio (ACR) was significantly higher at D14 FSGS, with a significant decrease by D28 of FSGS.

Podocyte number, identified by p57 staining, was abruptly depleted on day (D) 14 of FSGS by 39.6% from baseline to 6.5 per glomerular cross section (p = 0.025 vs. baseline) (Fig 1A–1D). Podocyte number per glomerular cross section was higher by day 28 FSGS compared to D14 (8.9 ± 2.5 vs.6.5± 1.38, p = 0.215 vs. D14 FSGS), consistent with partial replacement, as reported in other strains in this experimental model [8, 31, 32]. The changes in p57 staining was due to podocyte depletion, because glomerular nuclei, detected by DAPI staining, decreased markedly in segments of glomeruli, consistent with their loss, rather than simply reduced p57 staining due to podocyte injury (S1 Fig). The mean glomerulosclerosis score was higher at D14 (1.037±0.18 vs. 0.14±1.04, p<0.0001 vs. baseline). On day 28, glomerulosclerosis score had decreased, but was still higher than baseline (0.614±0.25 vs. 0.14±1.04, p<0.002 vs. baseline) (Fig 1E). The urinary albumin to creatinine ratio increased by D14 (249.14±55.89 vs. 13.5±5.06, p<0.0001 vs. baseline), with levels lower at D28 compared to day 14 (178.5±37.18 vs. 249.14±55.89, p = 0.016 vs. D14 FSGS) (Fig 1F). These results show that these characteristic features of FSGS are similar in this mouse strain to others [15, 32, 33] with initial podocyte depletion and glomerulosclerosis, followed by partial recovery.

#### Multiclonal cells of the renin lineage are detected in the JGC at baseline and after FSGS induction

**Fig 2:**
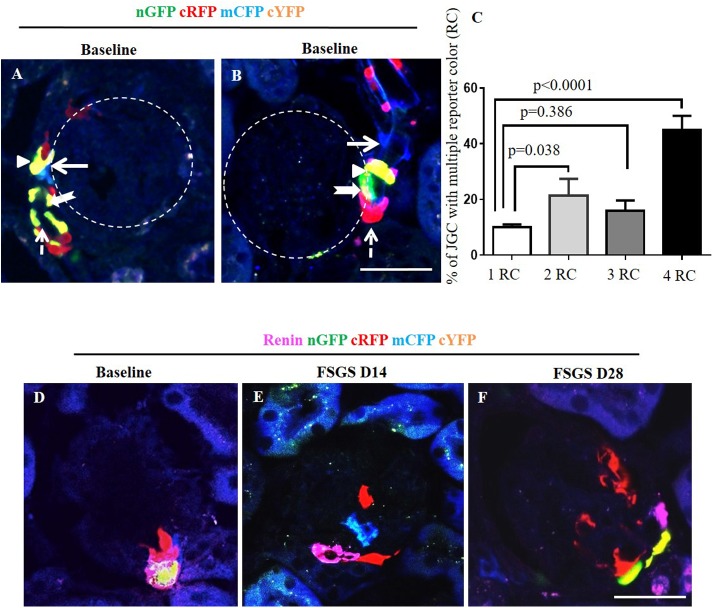
Confetti reporting in cells of renin lineage (CoRL) in normal and FSGS–induced *Ren1cCre /R26R-ConfettiTG/WT* mice with no evidence of CoRL proliferation. Frozen sections require no antibodies to detect the confetti reporters, nGFP (green), cRFP (red), mCFP (blue), and cYFP (yellow) while using confocal imaging. (A, B) Representative images from two different mice at baseline for nGFP (green, thick arrow), cRFP (red, dashed arrow), mCFP (blue, thick solid arrow), and cYFP (yellow, arrow head). All four CoRL reporter colors were detected only in the JGC, typically along afferent arterioles. Reporting was not detected in glomeruli, marked by the dashed white circles. (C) Graph showing that the majority of JGCs contain all four CoRL reporter colors (4RC) followed by two colors (2RC), three colors (3RC) and one color (1RC). The distribution of reporters was even, with no one reporter being dominant. (D) Representative image of baseline glomeruli showing co-localization of renin (magenta) and multi-clonal reporters. At D14 (E) and D28 (F) of FSGS, reporter labeled cells are detected on the glomerular tuft, which do not co-express with renin.

**Fig 3:**
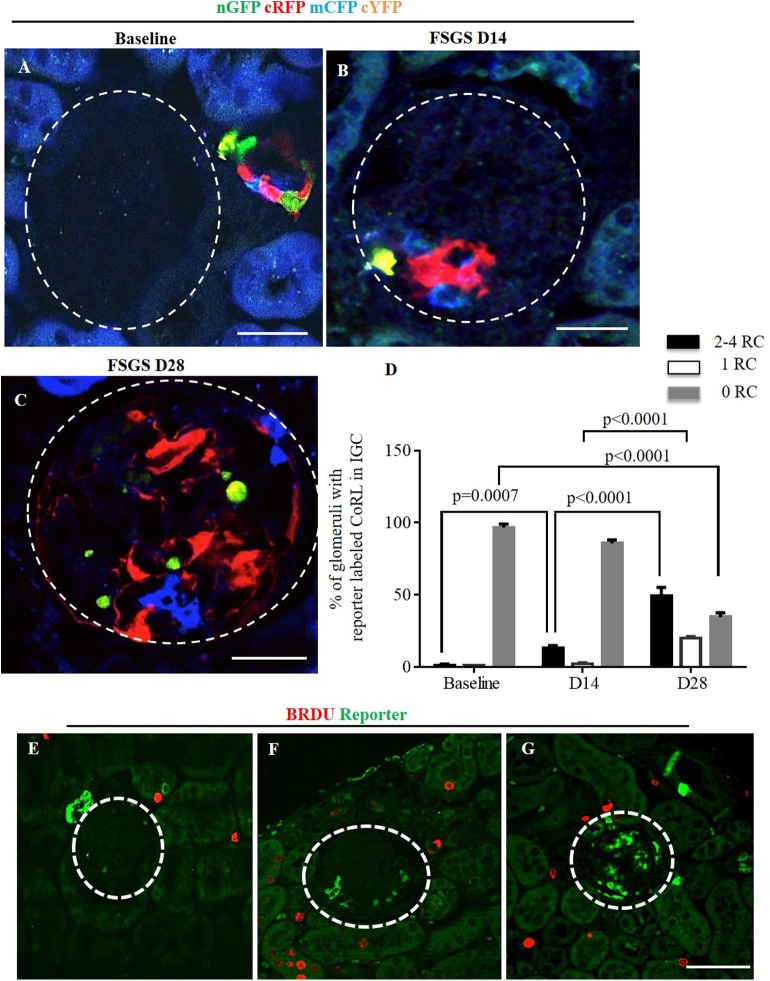
Multi-colored reporters of CoRL are detected in glomerular tufts of *Ren1cCre /R26R-ConfettiTG/WT* mice with FSGS. Confocal images showing four CoRL reporter colors detected without the use of antibodies–nGFP (green), cRFP (red), mCFP (blue) and cYFP (yellow). (A) All four reporters are restricted to the JGC at baseline, and are not detected in the glomerular tuft (dashed white circles). At D14 FSGS (B) and at D28 FSGS (C), all four CoRL reporter colors were detected in a subpopulation of cells in the glomerular tufts. (D) Graph showing that the percentage of glomeruli with reporter positive CoRL within the tuft was higher at D28 of FSGS and that these glomeruli contained 2–4 clones. (E) Represenative image showing all four reporters (converted to green color) and BRDU(red) do not co-localize at baseline. BRDU is present in some tubular epithelial cells as expected, but is not readily detected in JGC or the glomerular tuft. (F) BRDU staining increased at D14 of FSGS, but BRDU positive cells are not present in the JGC and glomerular tuft. (G) At D28 of FSGS there is an increase in the number of reporter labeled cells present on the glomerular tuft, however there is no overlap of reporters with BRDU.

Using mice harboring conditional or constitutive reporters, we showed that cells of renin lineage migrate from the juxta- to the intra-glomerular compartment following both acute [12] and chronic [34] podocyte depletion. In the current studies, we generated a *CoRL/Cre/confetti* mouse (*Ren1cCre/R26R-ConfettiTG/WT)* to determine if the CoRL progenitors were of mono- or poly-clonal in origin. Results shown in Figs 2 and 3 indicated CoRL are multiclonal.

Renin expression accompanies the development of the entire pre-glomerular vascular tree but is transient except for cells in the juxta-glomerular complex (JGC). Reporter detection did not require the use of antibodies, as each reporter was readily detected using confocal microscopy on frozen tissue sections. At baseline prior to disease induction, typically JGC contained different cells with all four CoRL reporter colors (GFP, YFP, CFP RFP) along afferent arterioles (Figs 2A, 2B and 3A). The percentage of JGC containing cells with either one, two, three or all four of the different reporters is shown in Fig 2C. A higher percent of JGC contain cells expressing two reporter colors (21.3±6.0% vs. 10.0±1.0%, p = 0.038), three colors (16±3.6% vs. 10.0±1.0%, p = 0.386) or all four colors (45.2±5.0% vs 10.0±1.0%, p<0.0001) of the reporter colors versus a single color. This finding indicated that even if bi-colored cells (red/blue or green/yellow) were observed as a result of continuous renin expression, at least two reporter colors were present in the majority of JGC, indicating mostly non-clonal populations of CoRLs. In majority of glomeruli, there were no reporter labeled cells, nor renin stained cells in the intra-glomerular compartment (IGC). However, there was overlapping staining with renin in cells in the JGC, consistent with reporter labeled cells expressing renin at baseline in JGC (Fig 2D).

Following podocyte depletion in experimental FSGS, reporter labeled cells were detected in the IGC. The absence of renin staining in these IGC reporter positive cells (Fig 2E and 2F) suggests that CoRL appear to migrate from the JGC to the IGC (Fig 3B and 3C) On D28 FSGS the percentage of glomeruli with absent reporter colors in intra-glomerular compartment (IGC) decreased significantly compare to baseline (34.67±5.05 vs. 96.67±4.16 p<0.0001 vs. baseline). Quantification of the percentage of glomeruli containing multiple reporter color cells (2–4 reporter colors) in the IGC, was 12 fold higher on D14 of FSGS compared to baseline (13.3±1.5 vs. 1.1±0.6, p = 0.0007) and another 3.7 fold higher on D28 FSGS compared to D14 (49.33±5.90 vs. 13.33±1.53, p<0.0001 vs. D14 FSGS). The percentage of glomeruli with a single reporter color in the IGC increased significantly on D28 FSGS compare to D14 (20.0±1.0 vs. 2.17± 0.76, p<0.0001 vs. D14 FSGS) (Fig 3D).

### CoRLs do not proliferate in the intra-glomerular compartment

BrdU staining was not detected in cells of JGC and IGC at baseline (Fig 3E). This was not a false negative, because BrdU was detected in occasional tubular epithelial cells as expected. At D14 of FSGS, reporter labeled cells in glomerular tuft did not stain for BrdU (Fig 3F). Likewise, on D28 of FSGS, BrdU staining was not detected in the glomerular tuft **(**Fig 3G).

### Multi-clonal CoRL reporters in a glomerular location co-express podocyte proteins

**Fig 4:**
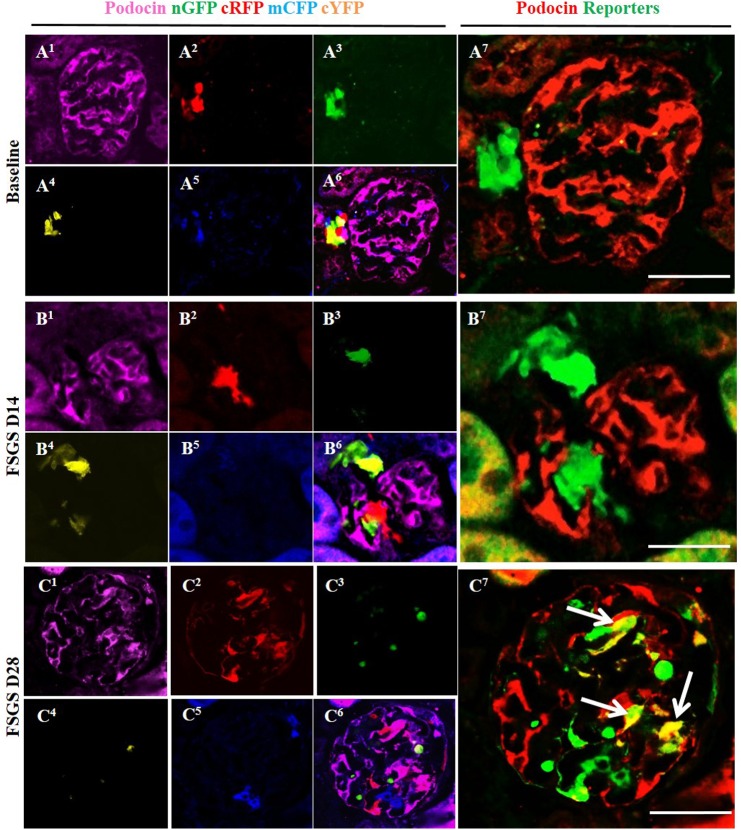
Labeled cells of renin lineage (CoRL) co-express podocin in glomeruli of *Ren1cCre /R26R-ConfettiTG/WT* mice with experimental FSGS. (A^1^-A^7^) At baseline: (A^1^) Confocal image shows podocin antibody staining (magenta). (A^2^-A^5^) All 4 CoRL reporters (green, red, blue, yellow) can be detected without the use of antibody. (A^6^) Composite image of all 4 reporters and podocin staining. (A^7^) For ease of viewing, all 4 confetti reporter channels have been converted to green, and podocin has been converted to red, so that co-localization can be visualized as yellow. All four confetti reporters are seen in the JGC, with no overlap with podocin staining. (B^1^-B^7^) At day 14 FSGS: (B^1^) There is a segmental decrease in podocin staining in the left lower quadrant of the glomerular tuft. (B^2^-B^6^) Multi-clonal CoRL (red, yellow and green) are detected in glomerular tuft, but do not co-localize with podocin. (B^7^) The CoRL reporters in the tuft do not co-localize with podocin staining. (C^1^-C^7^) At D28 FSGS: (C^1^-C^5^) Podocin and multi-clonal CoRL (red, yellow and green) are detected in the glomerular tuft. (C^6^) Composite image of all four reporters and podocin staining. (C^7^) CoRL reporters co-localize with podocin, creating a yellow color (arrows indicate examples).

**Fig 5:**
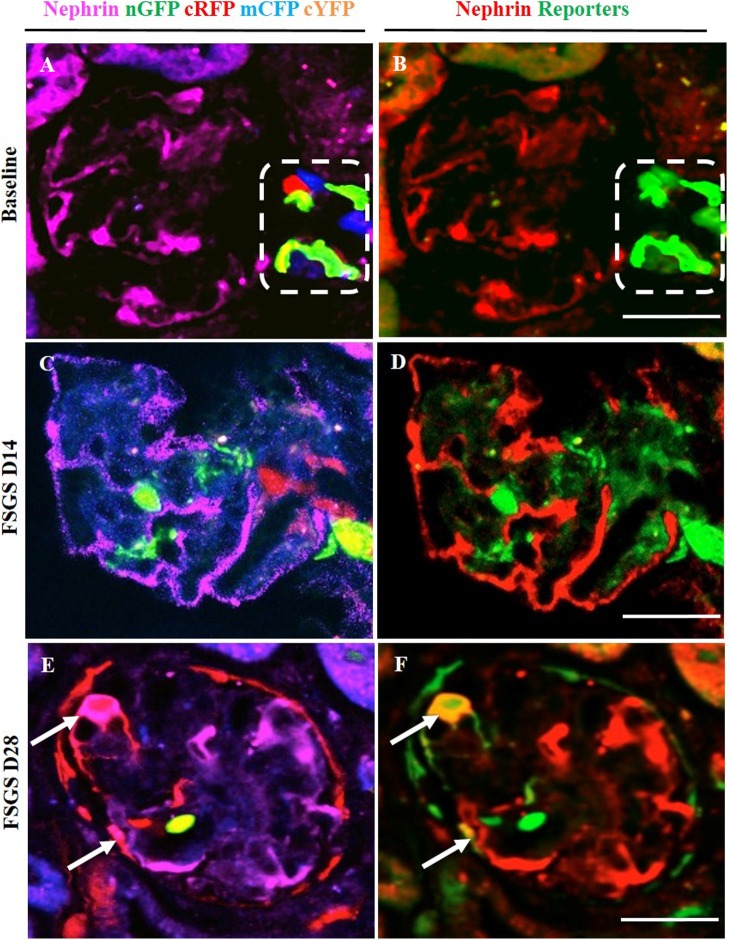
Labeled cells of renin lineage (CoRL) co-express nephrin in glomeruli of *Ren1cCre /R26R-ConfettiTG/WT* mice with experimental FSGS. The confocal images in the left column (A-C) represent nephrin staining (magenta) detected by antibody, and 4 CoRL reporters (green, red, blue, yellow) detected without antibody. The confocal images in the right column represents the same image on the left, but for ease of viewing, all 4 confetti reporter channels have been converted to green, and nephrin has been converted to red, so that co-localization can be visualized as yellow. (A, B) At baseline: All four CoRL reporter colors are restricted to the JGC (dashed white box), and nephrin staining is restricted to the glomerular tuft. (B) all four Confetti CoRL reporters (green) are seen in the JGC, with no overlap with nephrin (red). (C, D) At D14 FSGS: (C) There is a segmental decrease in nephrin staining in the right upper quadrant of the glomerular tuft. Multi-clonal CoRL are detected in glomerular tuft, but do not co-localize with nephrin. (D) The CoRL reporters in the tuft do not co-localize with nephrin staining. (E, F) At D28 FSGS: (E) Multi-clonal CoRL are detected in the glomerular tuft. (F) CoRL reporters co-localize with nephrin, creating a yellow color (arrows indicate examples).

**Fig 6:**
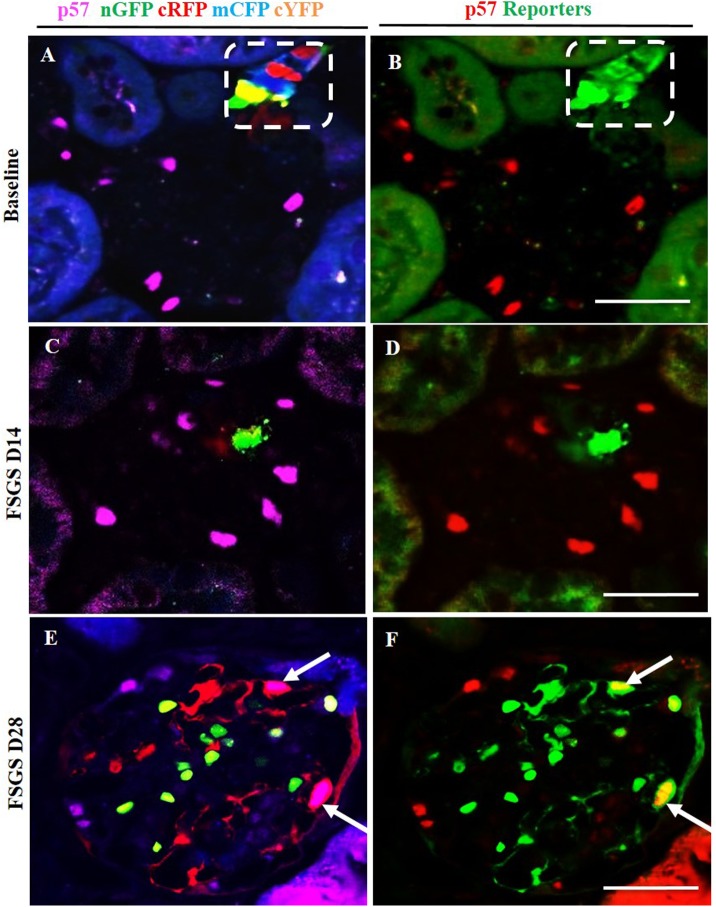
Labeled cells of renin lineage (CoRL) co-express p57 in glomeruli of *Ren1cCre /R26R-ConfettiTG/WT* mice with experimental FSGS. The confocal images in the left column (A-C) represent p57 staining (nuclear, magenta) detected by antibody, and 4 CoRL reporters (green, red, blue, yellow) detected without antibody. The confocal images in the right column represents the same image on the left, but for ease of viewing, all 4 confetti reporter channels have been converted to green, and p57 has been converted to red, so that co-localization can be visualized as yellow. (A, B) At Baseline: all four CoRL reporter colors are restricted to the JGC (dashed white box), and p57 staining is restricted to the glomerular tuft. (B) all four Confetti CoRL reporters (green) are seen in the JGC, with no overlap with p57 (red). (C, D) At D14 FSGS: (C) There is a segmental decrease in p57 staining in the right upper quadrant of the glomerular tuft. (D) The CoRL reporters in the tuft do not co-localize with p57 staining. (E, F) At D28 FSGS: (E) Multi-clonal CoRL are detected in the glomerular tuft. (F) CoRL reporters co-localize with p57, creating a yellow color (arrows indicate examples).

**Fig 7:**
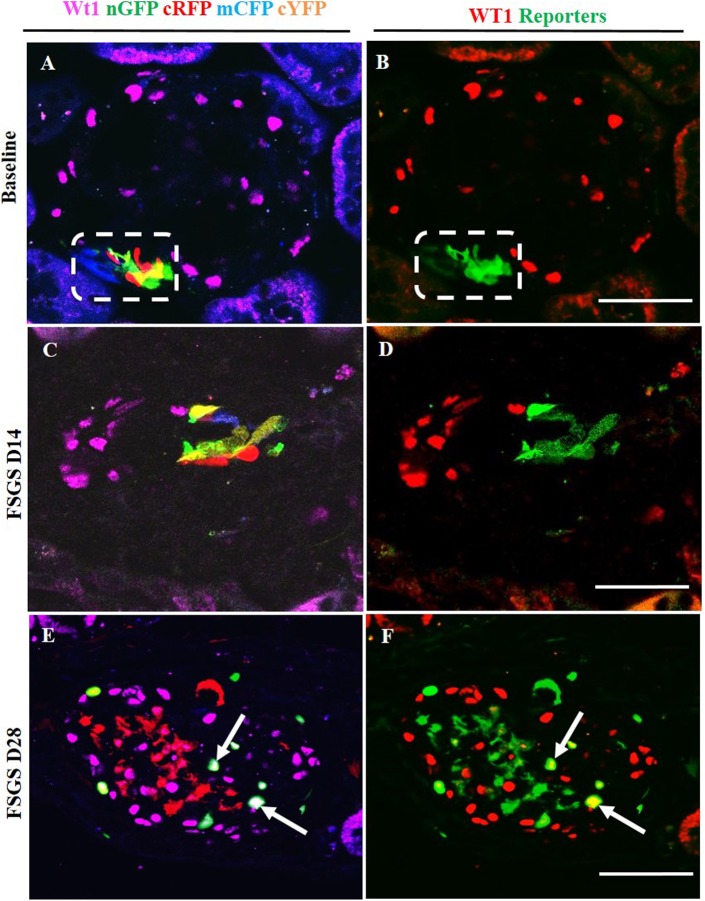
Labeled cells of renin lineage (CoRL) co-express WT-1 in glomeruli of *Ren1cCre /R26R-ConfettiTG/WT* mice with experimental FSGS. The confocal images in the left column (A-C) represent WT-1 staining (nuclear, magenta) detected by antibody, and 4 CoRL reporters (green, red, blue, yellow) detected without antibody. The confocal images in the right column represents the same image on the left, but for ease of viewing, all 4 confetti reporter channels have been converted to green, and WT-1 has been converted to red, so that co-localization is visualized as yellow. (A, B) At Baseline: (A) All four CoRL reporter colors are restricted to the JGC (dashed white box), and WT-1 staining is restricted to the glomerular tuft. (B) All four Confetti CoRL reporters (green) are seen in the JGC, with no overlap with WT-1 (red). (C, D) At day 14 FSGS: (C) There is a segmental decrease in WT-1 staining in the lower half of the glomerular tuft. Multi-clonal CoRL are detected in glomerular tuft, but do not co-localize with WT-1. (D) The CoRL reporters in the tuft do not co-localize with WT-1 staining. (E, F) At D28 FSGS: (E) Multi-clonal CoRL are detected in the glomerular tuft. (F) CoRL reporters merge with WT-1, creating a yellow color (arrows indicate examples).

To determine whether CoRL located in the intraglomerular compartment co-express podocyte proteins de novo in FSGS, double-staining was performed against the following four different podocyte proteins: podocin (Fig 4, membrane/cytoplasmic), nephrin (Fig 5, membrane/cytoplasmic), p57 (Fig 6, nuclear) and WT1 (Fig 7, nuclear). To improve the visualization of the overlap (Figs 4A^6^–4C^6^, 5A, 5C, 5E, 6A, 6C, 6E, 7A, 7C and 7E) between each of the CoRL reporter colors (Fig 4A^2-5^–4C^2-5^ and podocyte-specific proteins (Fig 4A^1^–4C^1^), all four reporter channels obtained on confocal microscopy were converted to appear green in color, and podocyte protein staining was converted to a red color, so that any co-localization created a yellow color (Figs 4A^7^, 4B^7^, 4C^7^ & 5–7F).

At D14 of FSGS, no reporter labeled CoRL present in glomeruli co-expressed the podocyte proteins podocin, nephrin, p57 or WT-1 (Figs 4B^6^, 4B^7^, 5–7C and 7D). In contrast, at D28 of FSGS, a subset of reporter labeled CoRL in diseased glomeruli co-expressed podocin (Fig 4C^6^ and 4C^7^), nephrin (Fig 5E and 5F), p57 (Fig 6E and 6F) and WT-1 (Fig 7E and 7F). These results suggest that a subset of multi-clonal reporter labeled CoRL present in the glomerular tuft co-express several proteins considered podocyte specific.

### Multi-Clonal CoRLs co-express PEC markers along Bowman’s capsule in FSGS

**Fig 8:**
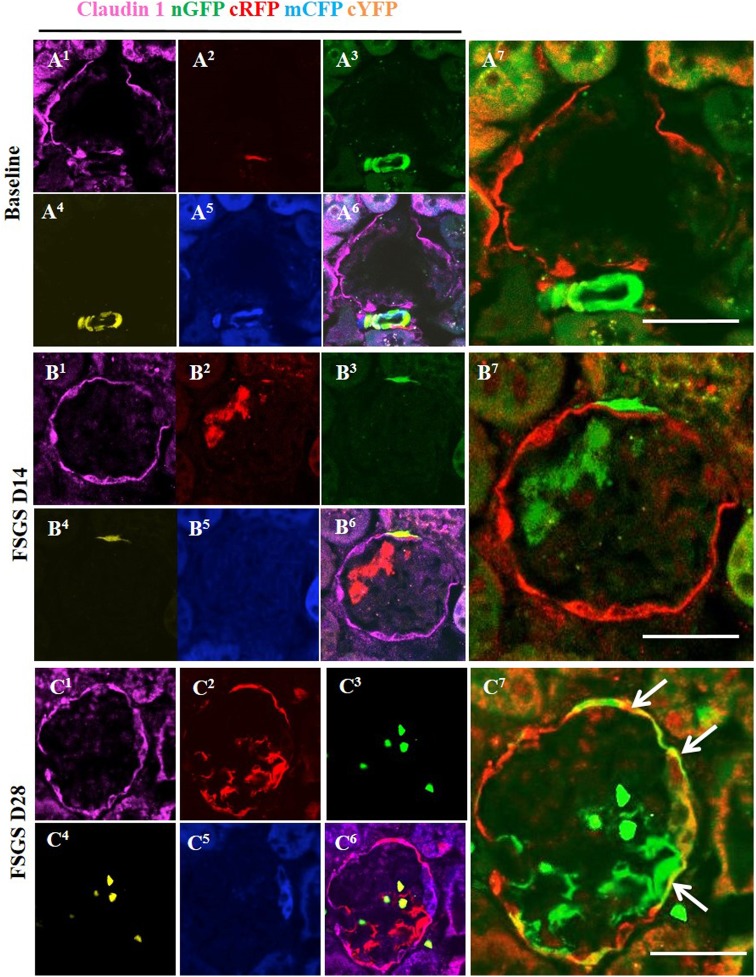
Multi-colored CoRL co-express the PEC protein Claudin-1 in FSGS. (A^1^-A^7^) At baseline: (A^1^) Representative confocal image showing claudin-1 staining (magenta) in its characteristic distribution. (A^2^-A^5^) All 4 CoRL reporters (green, red, blue, yellow) are restricted in JGC. (A^6^) Five-color composite image of reporters and Claudin1 staining. (A^7^) All four confetti reporters converted to green color are seen in the JGC, with no overlap with claudin-1 (red color). (B^1^-B^7^) At day 14 FSGS: (B^2^ –B^5^) Subset of reporter labeled cells (green) are detected along Bowman's capsule in close proximity to PEC’s. (B^6^, B^7^) However, CoRL reporters do not co-localize with claudin-1. (C^1^-C^7^) At day 28 FSGS: Multi-colored CoRL (C^2^-C^5^) are located along Bowman’s capsule and co-express claudin-1 (C^6^), creating a merged color (C^7^) (white solid arrows).

**Fig 9:**
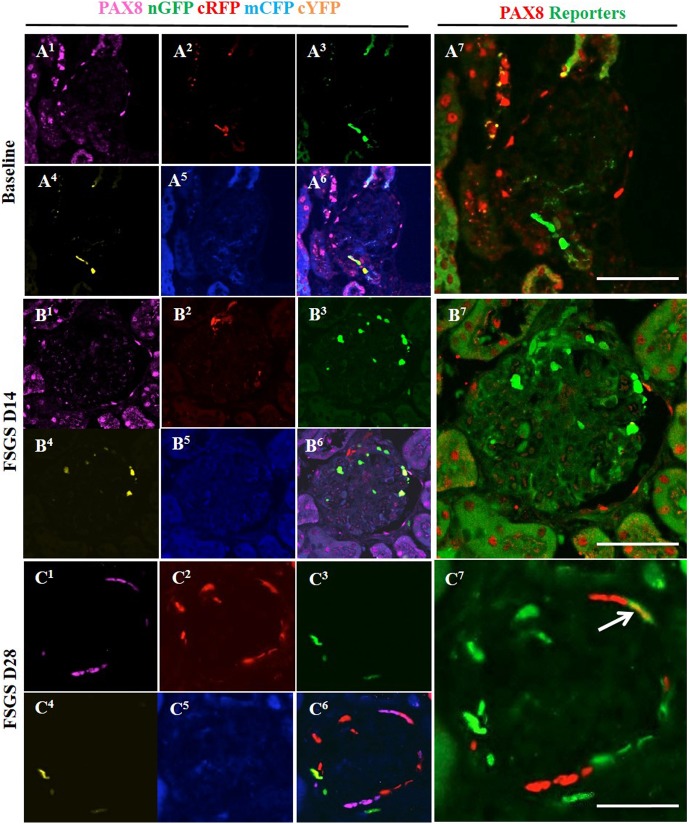
Multi-colored CoRL co-express the PEC protein PAX8 in FSGS. (A^1^-A^7^) At baseline: (A^1^) PAX8 staining is restricted to Bowman capsule (A^2^-A^5^) with no co-localization with multi-colored reporters (A^2^-A^7^). (B^1^-B^7^) At day 14 FSGS: There are no multi-colored reporter labeled CoRL (B^2^-B^5^) along Bowman's capsule that overlap with PAX8 staining. (B^1^). (B^6^-B^7^) Merged images of reporter and PAX8 staining. (C^1^-C^7^) At D28 FSGS: PAX8 staining (C^1^) co-localized with multi-colored reporter labeled CoRL(C^2^-C^5^) along Bowman’s capsule (C^6^, C^7^) (white solid arrow).

We have reported that a subset of CoRL migrated to Bowman’s capsule in disease co-express PEC markers [31, 32]. In order to show that reporter labeled CoRL co-express PEC markers, staining for Claudin-1 (Fig 8A^1^–8C^1^, membrane) and PAX8 (Fig 9A^1^–9C^1^, nuclear) were performed in *Ren1cCre/R26R-ConfettiTG/W*T mice. At baseline all four reporters were located in JGC (Figs 8A^2^–8A^5^ and 9A^2^–9A^5^) as expected, and did not show co-localization with the PEC markers Claudin-1 (Fig 8A^6^ and 8A^7^) and PAX8 (Fig 9A^6^ and 9A^7^). At day 14 of FSGS no reporter labeled cells that were observed along Bowman capsule (Figs 8 and 9B^2^–9B^5^) co-localized with PEC markers (Figs 8 and 9B^6^ and 9B^7^). At day 28 of FSGS a subpopulation of multi-colored CoRLs (Figs 8 and 9C^2^–9C^5^) along Bowman’s capsule co-expressed the PEC markers Claudin-1 (Fig 8C^6^ and 8C^7^) and PAX8 (Fig 9C^6^ and 9C^7^). These results show that in *Ren1cCre/R26R-ConfettiTG/WT* mice with FSGS, a subset of multi-clonal CoRL were observed along Bowman’s capsule and a subpopulation co-express PEC proteins.

### Serial intravital multiphoton microscopy imaging of *Ren1d-Confetti* mouse kidneys

**Fig 10:**
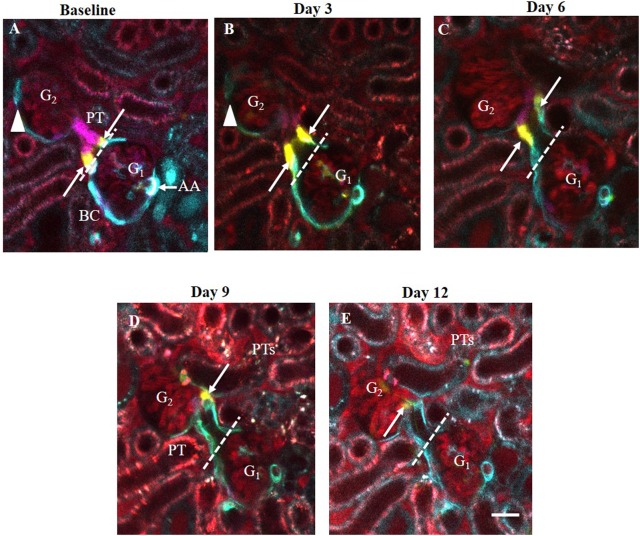
Serial intravital MPM imaging of the same glomeruli (G_1_-G_2_) in the same *Ren1d-Confetti* mouse kidney over two weeks after IgG-induced podocyte injury. (A) At baseline, several multi-color CoRL form the terminal portion of afferent arteriole (AA, mostly expressing blue, membrane-targeted CFP), the parietal Bowman’s capsule and the early proximal tubule (PT). Bar is 20 μm. Note the 1–2 yellow-labeled (cytosolic YFP expressing) CoRL (arrows) localized exactly at the glomerulo-tubular junction (dashed line). (B-E) Three-to-twelve days after IgG injection, the same YFP+ cells in the same glomerulus (G_1_) moved continuously away from the glomerulo-tubular junction and deeper into the proximal tubule. During the same time, CFP+ cells along the parietal Bowman's capsule of the other glomerulus (G_2_) disappeared from Bowman's capsule (arrowhead) between D3-6 (B-C). Plasma was labeled red using Alexa594-albumin. Note the significantly increased albumin content in G and PT fluid and within PT cells in Day 9–12 (intense red).

To understand the pattern and dynamics of glomerular cell remodeling by CoRL after IgG-induced podocyte injury, we performed serial multiphoton microscopy (MPM) of the same glomerulus in the same *Ren1d-Confetti* mouse kidneys *in vivo*. Dorsal abdominal imaging windows were implanted before the start of experiments, which allowed non-invasive optical z-sectioning of the same intact kidney every 3^rd^ day for the first two weeks after cytotoxic IgG injection. Individual CoRLs were identified at the single cell level using the unique Confetti color (either membrane-targeted CFP, nuclear GFP, or cytosolic YFP or RFP). Changes in CoRL cell distribution within glomeruli was determined by comparing z-stacks acquired at each time point (S1–S5 Movies). Because of the slow cell migration and remodeling process, serial MPM imaging of the same glomerulus was performed intermittently (every 3^rd^ day) rather than continuously. Therefore, it couldn’t be established with absolute certainty that the exact same cell was followed throughout the experiment. However, the number and relative position of pre-existing single cells (e.g. single yellow cell between blue cells, S1–S5 Movies), and the overall CoRL cell distribution (registered at each time point based on the unique single cell identifier color) appeared to be identical between time points, strongly suggesting that indeed the same cells were captured and followed over time. Also, we never observed any single cell within the glomerulus switching colors between consecutive imaging sessions (S1–S5 Movies), again strongly suggesting that *renin/Cre* were inactive in intra-glomerular CoRL. In addition, intravital MPM provided visual confirmation of the classic signs of podocyte injury and FSGS development after IgG treatment, including glomerular albumin leakage and increased proximal tubular albumin uptake (Fig 10D and 10E).

### CoRL-mediated remodeling of the PEC layer of Bowman’s capsule

We evaluated changes in CoRL distribution along the parietal epithelial cells (PEC) of Bowman’s capsule. We found that the position of select individual, CoRL-derived PECs was continuously shifting laterally away from the Bowman’s capsule into the early proximal tubule segment (Fig 10). The original position of PECs was taken by other CoRL that showed identical color with those PECs closer to the glomerular vascular pole and in the terminal afferent arteriole (blue, CFP-expressing in Fig 10), suggesting that the afferent arteriole provides a continuous supply of cells that differentiate into and migrate along the PEC layer. In the case of CoRL-derived PECs, the speed of lateral cell migration was very slow, approx. 20 μm over 3 days. This was measured by the change in the relative position of a single cell between imaging sessions that was assumed to be the same cell (Fig 10). Also, evidence was found for some CoRL disappearing from the PEC layer of Bowman’s capsule (Fig 10A–10C) suggesting that cell types other than the CoRL may also act as PEC precursors.

### Migration of CoRL to the visceral layer of the Bowman’s capsule

**Fig 11:**
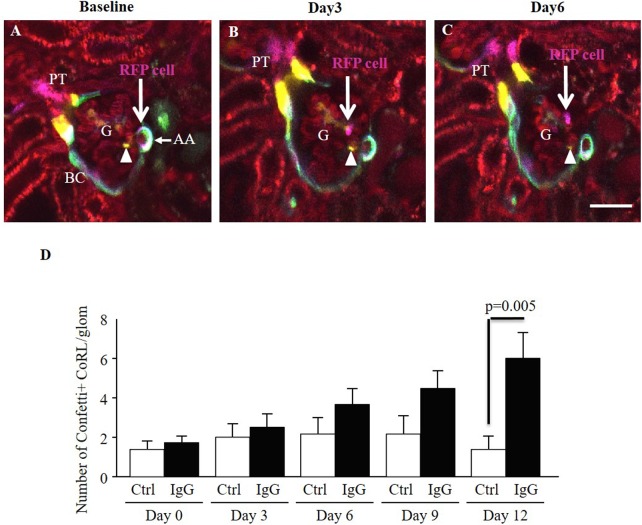
Tracking the migration of individually labeled single CoRL cells in the same *Ren1d-Confetti* mouse kidney after IgG-induced podocyte injury using serial intravital MPM imaging. (A) At baseline, several multi-color CoRL surround the terminal portion of the afferent arteriole (AA), line the parietal Bowman’s capsule and the early proximal tubule (PT). Bar is 20 μm. Note the magenta-labeled (cytosolic RFP expressing) single CoRL at the terminal AA closest to the glomerular tuft (RFP cell, arrow). (B) Three days after IgG injection, what is likely the same RFP+ cell in the same glomerulus (G) appears detached from the AA and localizes in the glomerular tuft (arrow). (C) Six days after IgG injection the RFP+ cell appears localized around a glomerular capillary (arrow). In contrast to the migrating RFP+ cell, a YFP+ CoRL (yellow, cytosolic YFP expressing) appears stationary in the intraglomerular mesangium (arrowhead). Plasma was labeled red using Alexa594-albumin. (D) Analysis of the total number of Confetti+ CoRL in the glomerular tuft in time-control (Ctrl, n = 5) and IgG-injected (IgG, n = 4) mice during the first two weeks of podocyte injury.

Serial intravital MPM imaging was able to track the migration of individually labeled single CoRL into the visceral layer of the Bowman’s capsule in intact living kidneys. Fig 11A–11C illustrates that cells which belong to the CoRL appear to detach from the terminal afferent arteriole compartment and migrate into the glomerular tuft, including locations in the mesangium and outside the glomerular capillary. This pattern and direction of CoRL migration may take several days (Fig 11A–11C). Importantly, the overall number of CoRL observed in the glomerular tuft occupying either mesangial and visceral Bowman’s capsule positions increased approximately 5-fold in response to podocyte injury compared to both baseline and timed controls over the first two weeks after IgG treatment (6.0±2.7 vs. 1.0± 1.5, p = 0.005 vs. control D12 FSGS) (Fig 11D).

### Persistence of CoRL-derived glomerular cell clusters

**Fig 12:**
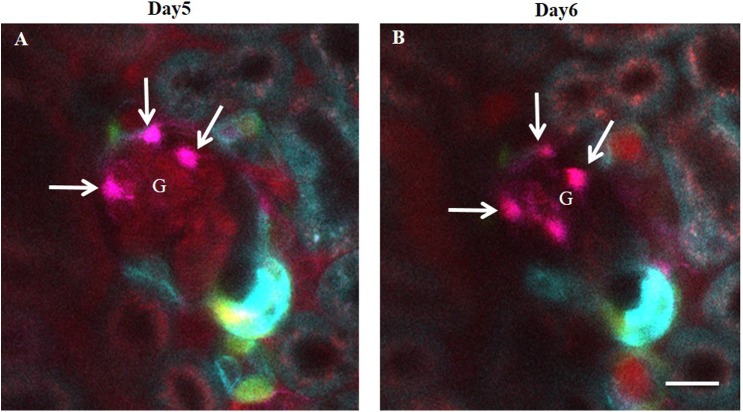
The formation of persistent CoRL-derived glomerular cell clusters after IgG-induced podocyte injury in *Ren1d-Confetti* mice. (A-B) Confetti+ cells (magenta, cytosolic RFP expressing) appear on the outside of glomerular capillaries 5 days after IgG injection (arrows) that resemble podocytes. Note the presence of several primary cell processes around capillaries. (B) Serial MPM imaging of the same glomerulus one day later showing the continued presence of same cells at the same locations. Plasma was labeled red using Alexa594-albumin. Bar is 20 μm.

Next, we addressed the persistence of CoRL recruitment to the glomerular tuft. Several clusters of CoRL were observed both along the parietal Bowman’s capsule (Fig 10) and surrounding glomerular capillaries (Fig 12). The cells on the outside of glomerular capillaries featured large, canopy-shaped cell bodies and several long cell processes wrapping around the capillaries, suggesting that they were differentiated podocytes (Fig 12A). Further support was that the same cells persisted at the same anatomical location for days (Fig 12A and 12B).

## Discussion

Replacement of glomerular cells, in particular terminally differentiated podocytes, is essential for the return of normal glomerular function following disease. Although studies are ongoing to determine the extent of progenitor involvement in these processes, the ability to study the clonality and movement of these populations has recently been made possible with the use of cell fate mapping strategies using multi-colored reporters and serial imaging of live cells. In the current study we show that following podocyte injury, cells of the renin lineage (CoRL) populate the glomeruli, and adopt phenotypic characteristics of both glomerular epithelial cell types namely podocytes and parietal epithelial cells (PECs).

These findings agree with previous work from several groups including ours and indicate that CoRL have remarkable plasticity, with the ability to change fate towards at least three glomerular cell types in disease: podocytes, PECs and mesangial cells [11, 15–17]. Yet, and until now, it was not known whether the CoRL originated from one cell in particular, or from multiple cells. To answer this fundamental question, we used reporter mice whereby cells of the renin lineage can be fate mapped stochastically by random selection of one of four colors. We found that CoRL which populated the glomerulus following abrupt podocyte depletion presented a mosaic pattern displaying 4 distinct colors. The results strongly indicate that during glomerular injury, cells from the renin progeny do not originate from a single dividing cell or clone but are instead multiclonal in origin. Although an inducible reporter system was not used, renin staining in both health and disease was limited to cells in the juxta-glomerular compartment, and was not detected in the glomerulus in disease in the current studies. This is similar to what we have reported for renin protein and mRNA expression in inducible renin reporter mice in this model [15]. These findings suggest that once in the glomerular compartment, cells of the renin lineage stop making renin and differentiate into other cell types as previously shown during normal development and glomerular disease [11, 17]. Therefore, despite studies not being in an inducible reporter system, the cessation of renin expression in the cells that migrate into the glomerular compartment would preclude *de novo* reporter expression resulting in the four distinct colors that were found within the glomerulus.

Using multiple injections of BrdU, we next asked if the presence of multiple clones of CoRL in diseased glomeruli was due to their proliferation within the glomerulus. The results showed that there were BrdU labeled cells in the tubules as expected. However, in the few BrdU labeled cells near the glomerulus, staining was restricted to labeled (and unlabeled) CoRL in the juxta-glomerular compartment, with barely any BRDU labeled cells detected within the glomeruli at the time points studied. This is consistent with our previous report [15]. We interpret these findings to indicate that the presence of multiple clones of CoRL in glomeruli is not the consequence of cell proliferation but likely from the movement of multiple individual cells from the juxta-glomerular compartment. This interpretation does not exclude the possibility, however, that local intraglomerular or glomerular parietal epithelial cells that derive from cells that transiently expressed renin in the early embryo may have also contributed to the regeneration of injured glomeruli. To be sure, similar studies would have to be performed using lineage tracing of podocytes and/or PECs. In fact, the relative contribution of each cell type to the regeneration of the injured glomerulus remains to be discerned, as multiple cell lineages may contribute to glomerular repair.

We next asked if all clones had the ability to co-express podocyte proteins. Indeed, the results showed that CoRL labeled with YFP, GFP, RFP and CFP all had the ability to co-express the podocyte markers podocin, nephrin, p57 and WT1. These results also support recently published work that CoRL serve an adult podocyte progenitor niche [13, 15, 28]. Similarly, multiple clones of labeled CoRL co-express the PEC markers claudin-1 and PAX8, consistent with the notion that they too serve as adult PEC progenitors in disease. The latter has also been shown during normal glomerular development [22, 35]. Taken together, both glomerular epithelial cell types could derive from multiple clones of CoRL following abrupt podocyte depletion. Nevertheless, as mentioned above, additional fate mapping with specific inducible cre lines will need to be performed to discern whether, and to what extent if any, PECs and/or remaining resident podocytes themselves contribute to repopulate the injured glomeruli.

Although fate mapping is used as proof for the migration of a labeled cell from one kidney location to another, an important question remained regarding whether CoRL migrate from the juxta- to the intra-glomerular compartment. Accordingly in these experiments we used a powerful complementary strategy, a recently developed direct visual approach to track the fate of fluorescently labeled glomerular cells in the intact living kidney using serial MPM imaging [22, 26]. The use of the multi-color Confetti reporter allowed us to label and identify individual CoRL and to perform genetic fate tracing in combination with serial MPM. It should be noted that although our Cre/lox-based experimental approach used a constitutively active Cre model (Ren1d-Cre), which labels cells that expressed renin in the early embryonic kidney and their descendants (even if actual renin expression has ceased), the combination with serial MPM imaging alleviated the potential technical issues.

Direct visualization of the CoRL cell population in the same glomerulus over time provided direct visual clues for the first time, suggesting the migration of individual CoRL from the terminal afferent arteriole to the parietal Bowman’s capsule and early proximal tubule, as well as to the glomerular tuft including the mesangium and the visceral layer of Bowman’s capsule. It should be emphasized that serial MPM imaging of the same glomeruli was performed intermittently rather than continuously over two weeks, therefore the identification of the same cell cannot be established with absolute certainty. This technical limitation was due to the rather slow process of CoRL migration and glomerular cellular remodeling. However, this experimental design was necessary to avoid cumbersome technical issues with continuous anesthesia, intravenous fluids and feeding, and significant cell damage due to laser exposure that would have been necessary and unavoidable with continuous MPM imaging. We believe that our serial MPM imaging approach provided strong visual clues regarding CoRL migration, and the new results represent a significant step forward in understanding the novel functions of CoRL. We feel confident that due to the advantages of the multi-color Confetti reporter and serial MPM approach, we indeed tracked the fate and migration of the same CoRL. Although it cannot be ruled out, we find it highly unlikely that another cell of the exact same Confetti-color belonging to the same CoRL lineage appeared in the exact same anatomical position at the exact same time.

We recognize limitations in the current study. First is the use of a constitutive reporter, which opens the possibility of cre-mediated recombination within podocytes and PECs following injury, due to activation of the *Ren1c* or *Ren1d* promotors in the two mouse strains used. We believe this is unlikely in the absence of any renin staining in podocytes or PECs. Furthermore, we have previously shown no *Ren1c* promotor activity in podocytes following injury using *Ren1cGFP* reporter mice [36]. Although not immediately feasible, it would be ideal to genetically pulse-label cells once, using an inducible system or perform single cell injection of intravital dyes before the injury and then track the cell movements along the chosen nephrovascular units. While those experiments are being contemplated, the results presented here in aggregate represent a significant advance and are consistent with the hypothesis that regeneration/repopulation of injured glomeruli may occur with the participation of CoRL that may reach the intraglomerular compartment from the juxta-glomerular compartment. Second, as stated above, the relative participation of PECs or local intraglomerular podocytes to the increase in podocyte mass after glomerular injury remains to be determined. Third, the experimental model used has partial podocyte replacement, but does not fully recover podocyte number to baseline levels following depletion.

Select CoRL appeared to differentiate into podocytes and remained at the same position around the glomerular capillary for several days. The increased number of CoRL recruited to the glomerular tuft after cytotoxic IgG injection suggests that podocyte injury triggers or augments glomerular remodeling by CoRL. These results are consistent with the previously established distribution of CoRL, which includes the glomerular afferent arterioles, mesangium, the parietal layer of Bowman’s capsule as well as various tubule segments and interstitium [10, 11, 19, 20]. Also, the relatively low number of CoRL observed in the present study in the glomerular tuft after podocyte injury is in good agreement with our previous work [13, 31].

In summary, we have used molecular genetic fate map reporting and live imaging approaches to better understand CoRL clonality during podocyte replacement and to monitor the fate of CoRL over time in the intact living kidney in experimental FSGS. Our data suggest that multi-clonal CoRL may migrate from the juxta-glomerular compartment and replace a subset of podocytes and PECs in experimental FSGS. Further work will be needed to determine whether the participation of CoRL and/or other cell types to the morphological repair results in lasting functional improvement of affected glomeruli.

## Supporting information

S1 Fig In *Ren1cCre /R26R-ConfettiTG/WT* mice with FSGS, podocyte loss results from cell loss. (A) Confocal image of DAPI (blue) showing a normal distribution of nuclei within the glomerulus (dotted circle) (B, C) Two representative images of D14 FSGS, showing segmental decrease in cell nuclei (marked with white lines) indicating cell loss. (D) Representative image of D28 FSGS with increased number of cell nuclei on the glomerular tuft (dotted circle), indicating cell replacement.
(DOCX)

S1 Movie of glomerular area in the same intact living kidney at the indicated time points over two weeks of serial MPM imaging. *Ren1d-Confetti* cells (cytosolic YFP expressing) attach to the terminal afferent arteriole compartment of bottom glomerulus. *Ren1d-Confetti* (CFP expressing) cells are presented along the parietal Bowman's capsule of the top glomerulus at Baseline.
(MOV)

S2 Movie showing that *Ren1d-Confetti* cells (cytosolic YFP expressing) detached from the afferent arteriole compartment of bottom glomerulus and observed in the glomerular tuft at D3 of FSGS. (MOV)

S3 Movie showing that *Ren1d-Confetti* cells (cytosolic YFP expressing) moved deeper into the proximal tubule compartment of the bottom glomerulus. *Ren1d-Confetti* (CFP expressing) cells are disappeared from the Bowman's capsule of the top glomerulus at D5 of FSGS.
(MOV)

S4 Movie showing that *Ren1d-Confetti* cells (cytosolic YFP expressing) moved continuously away from the glomerulo-tubular junction. CFP expressing cells are disappeared from Bowman's capsule at D9 of FSGS.
(MOV)

S5 Movie showing that *Ren1d-Confetti* cells (cytosolic YFP and CFP expressing) migrate along the parietal Bowman’s capsule and proximal tubule compartment at D12 of FSGS (MOV)

## Abbreviations

CoRL: cells of renin lineage
FSGS: focal segmental glomerulosclerosis
PECs: parietal epithelial cells
WT1: Wilms tumor suppressor protein 1
GFP: nuclear green fluorescent protein
YFP: cytoplasmic yellow fluorescent protein
RFP: cytoplasmic red fluorescent protein
CFP: membrane cyan fluorescent protein
BrdU: bromodeoxyuridine
DAPI: 4',6-diamidino-2-phenylindole
JGC: juxta-glomerular compartment
IGC: intraglomerular compartment
MPM: multiphoton imaging
