# Supplementary material for: Tracking the stochastic fate of cells of the renin lineage after podocyte depletion using multicolor reporters and intravital imaging
Source: PLoS One. 2017 Mar 22;12(3):e0173891. doi: 10.1371/journal.pone.0173891 (PMC5362207; doi:10.1371/journal.pone.0173891)

**S1 Fig. In *Ren1cCre /R26R-ConfettiTG/WT* mice with FSGS, podocyte loss results from cell loss.** (A) Confocal image of DAPI (blue) showing a normal distribution of nuclei within the glomerulus (dotted circle) (B, C) Two representative images of D14 FSGS, showing segmental decrease in cell nuclei (marked with white lines) indicating cell loss. (D) Representative image of D28 FSGS with increased number of cell nuclei on the glomerular tuft (dotted circle), indicating cell replacement.


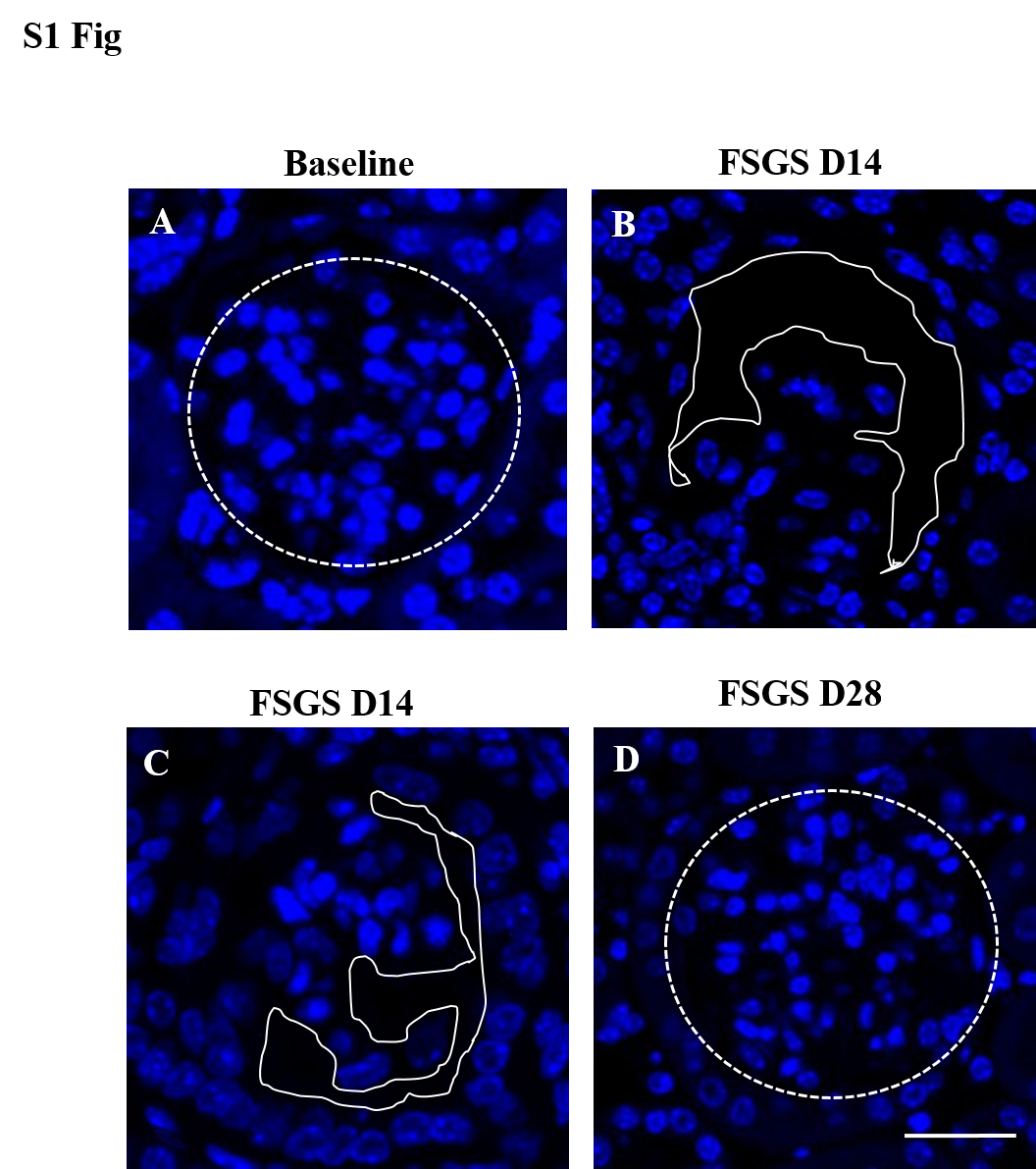

Supplement: S1 Fig — (A) Confocal image of DAPI (blue) showing a normal distribution of nuclei within the glomerulus (dotted circle) (B, C) Two representative images of D14 FSGS, showing segmental decrease in cell nuclei (marked with white lines) indicating cell loss. (D) Representative image of D28 FSGS with increased number of cell nuclei on the glomerular tuft (dotted circle), indicating cell replacement. (DOCX) [file pone.0173891.s001.docx]
